# Breast Tissue Biology Expands the Possibilities for Prevention of Age-Related Breast Cancers

**DOI:** 10.3389/fcell.2019.00174

**Published:** 2019-08-28

**Authors:** Tara Fresques, Arrianna Zirbes, Sundus Shalabi, Susan Samson, Sandy Preto, Martha R. Stampfer, Mark A. LaBarge

**Affiliations:** ^1^Department of Biological Systems and Engineering, Lawrence Berkeley National Laboratory, Berkeley, CA, United States; ^2^Department of Population Sciences, Beckman Research Institute at City of Hope, Duarte, CA, United States; ^3^Center for Cancer and Aging Research, Beckman Research Institute at City of Hope, Duarte, CA, United States; ^4^Medical Research Center, Al-Quds University, Jerusalem, Palestine; ^5^Breast Science Advocacy Core, Breast Oncology Program, University of California, San Francisco, San Francisco, CA, United States; ^6^Notes4Hope, Moraga, CA, United States

**Keywords:** breast cancer, prevention, chemoprevention, immortality, inflammaging, warfarin, metformin

## Abstract

Preventing breast cancer before it is able to form is an ideal way to stop breast cancer. However, there are limited existing options for prevention of breast cancer. Changes in the breast tissue resulting from the aging process contribute to breast cancer susceptibility and progression and may therefore provide promising targets for prevention. Here, we describe new potential targets, immortalization and inflammaging, that may be useful for prevention of age-related breast cancers. We also summarize existing studies of warfarin and metformin, current drugs used for non-cancerous diseases, that also may be repurposed for breast cancer prevention.

## Introduction

There are limited options for prevention of breast cancer. Tamoxifen, raloxifene, and aromatase inhibitors are currently used for breast cancer prevention in the recurrence setting and have been shown to be effective in large scale trials (Kinsinger et al., 2002). However, they are not used in low risk scenarios due to side effects such as deep vein thrombosis (Kinsinger et al., 2002). Epidemiological approaches to identify means to protect individuals from developing breast cancer have been heavily influenced by age and estrogen receptor status. More than 75% of breast cancers in the United States are diagnosed in women aged over 50 (Smigal et al., 2006; Jemal et al., 2007), and 80% of age-related breast cancers are hormone-receptor expressing luminal subtypes, whereas the triple negative disease is enriched among younger women (Jenkins et al., 2014). The dominant paradigm suggests that the higher incidence of age-related cancers is due to accrual of somatic mutations over time that alter regulation or activity of oncogenes and tumor suppressors (DePinho, 2000). A number of cancers show an exponential increase in incidence with age, consistent with the mutation accumulation hypothesis. However, the incidence of breast cancer decreases sometime after age 70 (Anderson et al., 2014). In addition, women from different countries, e.g., Japan versus United States, exhibit very different distributions for the age of first breast cancer diagnosis (Matsuno et al., 2007) despite both being industrialized nations with, we assume, similar mutation rates (Todhunter et al., 2018). Thus, this evidence does not support accumulation of mutations alone as an explanation of the age-related increase in breast cancer incidence. Examination of the cellular and molecular processes that underlie aging in the breast may reveal new avenues for breast cancer prevention.

A number of systemic changes occur in the breast as a result of age such as a significant decrease in estrogen production in the transition to and during menopause. These hormonal differences likely cause significant changes in the physical properties of breast tissue as many studies have found that hormone changes coincide with decreased connective tissue, increased adipose, and discontinuities in the basement membrane, which maintains normal polarity of the epithelium (Howeedy et al., 1990; Milanese et al., 2006; Well et al., 2007). Furthermore, significant changes occur as a result of age in mammary epithelial cells. For example, dysfunctional luminal-biased progenitors and luminal cells with acquired myoepithelial-like characteristics accumulate, whereas tumor-suppressing myoepithelial cells decrease in proportion (Garbe et al., 2012). These cellular changes may cause gradual functional changes at the level of tissue structure that can corrupt the tumor-suppressive activity of normal tissue architecture. These and other alterations lead to tissue-level phenotypes hypothesized to make older breast epithelia more susceptible to transformation (reviewed in LaBarge et al., 2016).

Furthermore, experiments with normal human mammary epithelial cells (HMEC) suggest that cells from older women have intrinsic qualities that pre-dispose them to develop the breast cancer subtypes that are more commonly found in older women. When normal HMEC from post-menopausal women are intentionally transformed to immortal states they exhibit gene and protein expression consistent with luminal breast cancer subtypes, whereas similarly treated cells from younger women exhibit properties consistent with a basal phenotype (Lee et al., 2015). Using heterochronus cell culture models of human mammary epithelia it was shown that the tissue microenvironment drives the age-related epigenetic and transcriptional phenotypes of the luminal epithelial lineage (Miyano et al., 2017). This suggests that age-related epigenetic states may underlie the prevalence of luminal subtype breast cancers among older women.

Aging also causes significant phenotypic changes in the putative breast cancer cells of origin, cKit-expressing luminal-biased epithelial progenitor cells (Lim et al., 2009). These cells acquire a basal differentiation bias with age (Garbe et al., 2012), due in part to gain in activity of the YAP transcription factor (Pelissier et al., 2014), which is known to provide access to epithelial-to-mesenchymal transition (EMT)-related programs (Shao et al., 2014). Intriguingly, the luminal-biased cKit-expressing epithelial progenitors that accumulate with age were shown to express a unique signature of signaling molecules (comprised of Axl, YAP, pS6, pPLCg2, pEGFR, CD44, and pGSK3), which is the same protein signature that emerges in immortal transformed luminal cells at the very earliest stages of cancer progression (Pelissier Vatter et al., 2018). Taken together, the aging process: (i) endows progenitor cells with features of early cancer, (ii) causes epigenetic changes in the epithelia that may underlie the types of breast cancers most commonly seen in older patients, and (iii) diminishes the ability of the tissue to resist malignant progression by eliminating the myoepithelial gate keepers.

We speculate that successful forms of breast cancer prevention would bolster processes that help maintain tissue integrity, such as forcing progenitors to differentiate into harmless terminal states, or decreasing the low-grade, chronic inflammation that accompanies the aging process, which is thought to precede many cancers. Alternatively, because cancer has a long preamble and aging appears to prime cells to enter early stages of malignant progression, targeting the transition states between normal and malignant may be done in the context of age-related breast cancers. In this review, we consider a number of possible biological targets that may be exploited for breast cancer prevention that span a continuum from theoretical, to drug repurposing, and even ongoing cancer prevention clinical trials. Indeed, it may be possible that common treatments for maladies that are often age-associated could be effective as chemoprevention for age-related breast cancers.

## Targeting the Transition to Immortality

Stopping cancer before it is able to form in susceptible breast cells would be an ideal way to prevent breast cancer in general, including age-related breast cancers. Many different molecular changes can propel normal mammary epithelial cells toward cancer; therefore a good first step for developing preventive strategies is to define the processes that propel progression. Ideally, a molecular process that exhibits the following qualities would provide an excellent target for breast cancer prevention:

(1) Occurs in all precursor cancer cells.
(2) Occurs prior to the acquisition of malignant properties and is required for malignancy.
(3) Does not occur in normal finite cells.
(4) Is unique to the process of oncogenesis and has limited-to- no parallel mechanisms that can achieve the same result.

**FIGURE 1:**
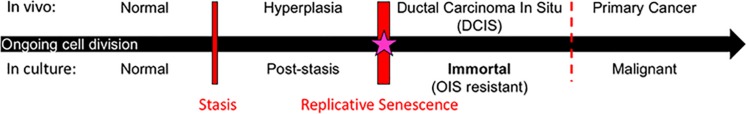
Model of breast cancer progression and barriers for potential chemoprevention targets. Normal cells continue to divide in culture until they approach the stress-associated stasis barrier; cells can bypass stasis by functional inhibition of the retinoblastoma pathway. Post-stasis cells continue to divide until they approach the replicative senescence barrier, which results from ongoing telomere erosion producing telomere dysfunction and genomic instability. Reactivation of telomerase in post-stasis cells can confer immortality. Eroded telomeres, genomic instability, and telomerase reactivation similarly occur at the DCIS stage *in vivo*. Our research suggests that immortalization coincides with a cancer-unique re-structuring of telomere maintenance mechanisms. Immortalized cells are then resistant to oncogene induced senescence (OIS) and many oncogenes can cause them to become malignant. We propose that the immortalization barrier can be a valuable target for breast cancer prevention (starred).

Studies to uncover processes involved in transitioning normal finite HMEC to malignancy have shown that two molecularly distinct barriers stop normal HMEC from gaining immortality, an essential step in early cancer progression (Figure 1) (Stampfer et al., 1997, 2003, 2013; Garbe et al., 2009, 2014; Lee et al., 2015). The first is a stress-associated senescence barrier (stasis). Cells need to inhibit the retinoblastoma pathway in order to bypass this stasis barrier and continue dividing (Garbe et al., 2009, 2014). The second barrier is replicative senescence due to critically short telomeres. Cells need to reactivate telomerase in order to overcome this barrier and become immortal (Garbe et al., 2009, 2014). The process involved in overcoming replicative senescence and becoming immortal may be an ideal target for breast cancer prevention as it meets the four criteria described above. (i) One of the defining characteristics of all cancer cells is their ability to proliferate indefinitely. Telomerase reactivation, which confers immortality, is thought to occur during the pre-malignant ductal carcinoma *in situ* (DCIS) stage of breast cancer progression (Chin et al., 2004; Meeker et al., 2004). Therefore, cancer cells achieve immortalization in their precursor population. (ii) Obtaining immortality is crucial for cells to become vulnerable to malignant transformation. This is due not just to obtaining unlimited proliferative capacity, but also due to oncogene-induced senescence, meaning that malignancy-causing oncogenes will only cause malignancy in cells that have attained immortality (Olsen et al., 2002), but in contrast, will cause finite cells to senesce and die (Olsen et al., 2002). Therefore, therapeutics that target breast cancer precursor cells before they become immortal could stop them from becoming malignant. (iii) Normal finite cells never undergo the cancer-associated immortalization process, thus normal cells should not succumb to a therapeutic targeted toward this process. (iv) The vast majority of human carcinoma cells use reactivation of telomerase to achieve immortality. While some cancers use a homology recombination-based mechanism, termed alternative lengthening of telomeres (ALT), to become immortal, this mechanism is rarely observed in breast and most other human carcinomas (Bryan et al., 1997; Shay and Bacchetti, 1997; Subhawong et al., 2009). Thus, if telomerase reactivation is inhibited for prevention purposes, cancer precursors do not have a ready parallel bypass mechanism to compensate. For these reasons, the process of telomerase reactivation during immortalization is a promising process to target for prevention of most human carcinomas.

The molecular mechanisms that cause the immortalization process are beginning to be uncovered and include two phenomena. First, post-stasis cells acquire an error permissive for expression of the telomerase gene and become conditionally immortal (Stampfer et al., 1997, 2003; Garbe et al., 1999). However, for sufficient telomerase activity to maintain stable telomeres, these cells need to undergo a successful second event that we have termed conversion (Stampfer et al., 1997, 2003; Garbe et al., 1999). The conversion process involves a change in telomere dynamics that occurs as a result of the initial immortalization-inducing error (Stampfer et al., 1997, 2003; Garbe et al., 1999). Notably, the mean telomere restriction fragment length (TRF) of immortalized HMEC lines and most human cancers is approximately 4 kb (Stampfer et al., 1997; Listerman et al., 2013; Barthel et al., 2017). This is in stark contrast to all normal finite cells in the human body whose mean TRF does not go below ∼5 kb (Harley et al., 1990; Aubert et al., 2012). We hypothesize that the conversion process involves a restructuring of telomeres to allow regulation that supports maintaining short stable telomeres, similar to what is seen in single-celled organisms such as yeast (Shore and Bianchi, 2009).

Future research that aims to understand the molecular features of the immortalization process will be valuable to develop prevention therapeutics. Ideally, research should start with normal finite cells that have been made post-stasis following molecular perturbations that are prevalent in most breast cancers. In order to induce and follow immortalization we have previously studied cell lines that became immortal following exposure to benzo(a)pyrene (Stampfer and Bartley, 1985; Stampfer et al., 1997). More recently we have been able to induce immortalization by transduction of post-stasis HMEC with a c-Myc transgene (Garbe et al., 2009, 2014; Lee et al., 2015). Research with these and other models have revealed some molecular features that may be unique to the immortalization process, such as loss of the long non-coding RNA MORT (Nijjar et al., 1999; Stampfer et al., 2003; Garbe et al., 2014; Lee et al., 2015; Vrba et al., 2015). Another intriguing target may be proliferating cell nuclear antigen (PCNA), which is thought to undergo a post-translational modification that is detected only in cancer and cancer precursor cells, as early as the DCIS stage (Gu et al., 2018). There are pre-clinical molecules known to target and kill cancer cells harboring this unique form of PCNA and thus represent a potential prevention agent that stops recently immortalized cells in their tracks (Gu et al., 2018). Therapeutics designed to inhibit the cancer-associated immortalization process may prevent a majority of breast cancers before they have a chance to form.

## Targeting Inflammaging to Reduce Susceptibility to Breast Cancer

The aging immune system is characterized by innate immune changes that include a type of chronic, low-grade, macrophage-centered, sterile inflammation known as inflammaging (Palmer et al., 2018). At a basic level, inflammation is an organized immune system response to infection or tissue injury in which several cell types and chemical signaling molecules are recruited to the site of injury and begin a process of wound-healing. The most common signaling molecules involved in inflammation, which are used as characteristic markers, include: tumor necrosis factor-alpha (TNF-α), transforming growth factor-beta (TGF-β), and interleukins-1, 6, and 18 (IL-1, IL-6, and IL-18) (Bonafe et al., 2012; Prattichizzo et al., 2016; Xia et al., 2016). Serum levels of IL-6 and C-reactive protein (CRP), are often used to assess inflammatory levels in patients (Barbaresko et al., 2013). Regulation of expression of pro-inflammatory cytokines and proper timing of expression of opposing anti-inflammatory cytokines during an immune response is needed for homeostasis. Over-expression of pro-inflammatory cytokines can lead to chronic inflammation and autoimmunity, and conversely over-expression of anti-inflammatory cytokines can lead to immune suppression.

Immune cells in normal breast tissue primarily localize to breast lobules, where they closely associate with the epithelium, rather than stroma or fat (Degnim et al., 2014). Murine mammary gland studies revealed the importance of immune-epithelial cell interactions that cause phenotypic and compositional changes in the mammary epithelia during development (Gouon-Evans et al., 2002; Lilla and Werb, 2010; Reed and Schwertfeger, 2010; Plaks et al., 2015). The composition and function of immune cell populations are known to change in peripheral blood with age (e.g., increased macrophages and dendritic cells, decreased T cells, and reduced function of cytotoxic T cells) (Plackett et al., 2004; Weiskopf et al., 2009), and in breast tissue during breast cancer progression (e.g., increased macrophages) (Ruffell et al., 2012; Degnim et al., 2017; Linde et al., 2018). How age-related changes in immune cell populations, and their effects on aged mammary epithelia, are relevant to increased breast cancer susceptibility with age is not well-understood.

The components of inflammaging that plausibly drive breast cancer initiation and progression include age-related DNA damage, cell senescence, and obesity. Increased DNA damage accumulation with age is a contributing factor to inflammaging. When mammary stem cells and stromal fibroblasts incur DNA damage, they secrete pro-inflammatory cytokines, including IL-6 and IL-8 that can affect surrounding cells (Dieriks et al., 2010; Ivanov et al., 2010). The cytokines in turn induce further DNA damage, cause alterations in the surrounding target cells, and recruit macrophages to the area leading to more inflammation. Inflammation further enables transformation of the surrounding cells, and inflammatory lymphocytes and macrophages are thought to accelerate transformation of mammary epithelia (Lin et al., 2006; Rao et al., 2006). Increases in senescent cells are synonymous with aging which is associated with the senescence-associated secretory phenotype (SASP), a phenomenon that causes senescent cells to activate an inflammatory transcriptional program. Senescence protects cells from transformation, but paradoxically, senescent cells secrete a number of pro-inflammatory cytokines and matrix metalloproteinases that act on neighboring cells in a deleterious manner to induce changes in gene expression that are associated with transformation (Krtolica et al., 2001; Coppe et al., 2010; Borodkina et al., 2018). There is a long-established correlation between the age-associated increase in obesity and breast cancer (Picon-Ruiz et al., 2017). One plausible link between obesity and breast cancer is the pro-tumorigenic and pro-angiogenic microenvironment generated by increased secretion of pro-inflammatory cytokines, like IL-6, by macrophages in adipose tissue (Seiler et al., 2018). Thus the release of pro-inflammatory molecules and microenvironment remodeling enzymes that result from cell and tissue changes that are associated with aging comprise a similar set of mechanisms that underlie the inflammaging phenomenon.

There is an overall association between chronic low-level inflammation and aging phenotypes in multiple tissues, however, the actual impact on aging phenotypes of mammary epithelia remains to be demonstrated. If there is a relationship between inflammaging in breast with deleterious epithelial changes and increased breast cancer susceptibility, then weight loss and anti-inflammation strategies would comprise the main thrust of a prevention approach. This could also include aspirin, which has been suggested to be preventive for breast cancer through an as-yet unknown mechanism (Clarke et al., 2017). In addition, a number of foods are considered anti-inflammatory and may reduce inflammaging, such as fruits, vegetables, fish, and whole grains (Barbaresko et al., 2013; Calder et al., 2017; Kaluza et al., 2018). Continued research examining the mechanistic link between inflammaging and breast cancer susceptibility may provide more useful therapeutic targets for prevention.

## Warfarin as a Putative Prevention Agent

Warfarin is commonly prescribed in Western countries for atrial fibrillation, venous thromboembolism, and a number of other cardiac-related indications. Although use is steadily declining in favor of newer anti-coagulants that have preferable safety profiles, warfarin remains one of the most heavily prescribed anti-coagulants with as many as seven million users in the United States as of 2014 (Barnes et al., 2015). A majority of warfarin users are over 60 years of age; thus this drug is particularly intriguing in the context of age-related breast cancer prevention. Epidemiological studies have identified a putative cancer prevention effect of warfarin in this older population in multiple cancer contexts. Women who used warfarin for at least 6 months showed 10–30% reduced relative risk of breast cancer compared to non-warfarin users (Schulman and Lindmarker, 2000; Tagalakis et al., 2007; Haaland et al., 2017). Similar anti-cancer effects were reported in animal models (Ryan et al., 1968; Williamson et al., 1980; Paolino et al., 2014), which also revealed that warfarin doses with no anti-coagulation activity also could be effective in a prevention context (Kirane et al., 2015), thus potentially avoiding some of the negative safety issues associated with warfarin use.

**FIGURE 2:**
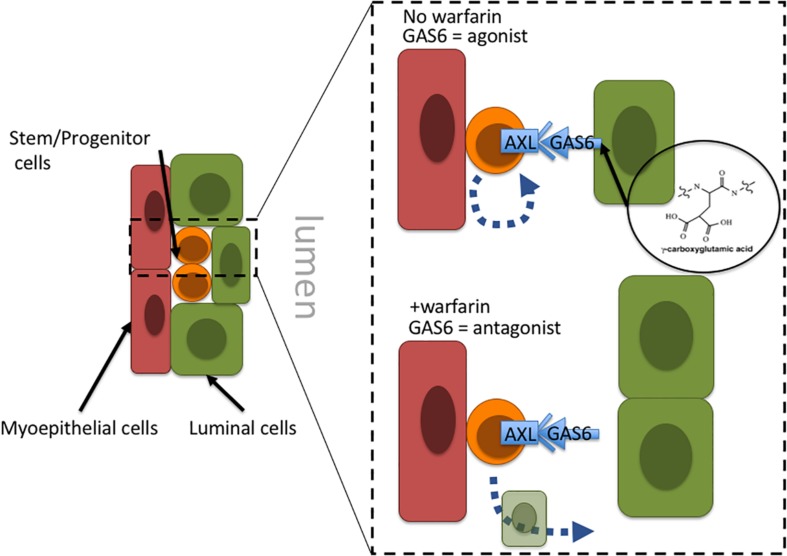
Proposal of a tissue-level mechanism of warfarin’s putative anti-breast cancer effects. In mammary epithelia Axl signaling allow cells with progenitor properties access to stem-cell gene programs; engagement with the GAS6 ligand maintains progenitors in an undifferentiated state. Warfarin inhibits gamma-carboxylation of the Axl ligand, GAS6, preventing it from remaining anchored in the plasma membrane and essentially converting GAS6 from an agonist to an Axl-antagonist. At that point the progenitors may differentiate into more terminal states. Because the epithelial progenitors are thought to comprise breast cancer cells of origin, it might be more advantageous to force them to differentiate before they become a liability.

Warfarin inhibits vitamin K oxidoreductases, resulting in depletion of vitamin K and non-carboxylated γ-carboxyglutamate domains of vitamin K-dependent proteins. Most of the ∼14 known proteins that are vitamin K-dependent are involved in coagulation of blood; however, growth arrest specific 6 (GAS6) and periostin (POSTN) also require γ-carboxylation. Haaland et al. (2017) hypothesize that in the absence of γ-carboxylation GAS6 cannot remain anchored in the plasma membrane and thus converts GAS6 from being an Axl receptor tyrosine kinase agonist into an antagonist. Inhibiting Axl has the impact of reducing malignant traits in aggressive mammary carcinomas, as well as increasing natural killer cell activity (Gjerdrum et al., 2010; Kirane et al., 2015). Axl signaling is linked to induction of epithelial-to-mesenchymal transitions in cancer cells, and induction of stem cell-like properties, suggesting an overall role in regulation of stem-like states (Vuoriluoto et al., 2011; Jokela et al., 2018). Although speculative, inhibition of Axl with an antagonist-form of GAS6 may prevent cancer stem cells from remaining in a stem-like state and instead allow them to differentiate into terminal states (Figure 2). Another potential target of warfarin, periostin (POSTN), is thought to improve cancer cell survival and, in some contexts, increase proliferation by increasing microenvironment stiffness due to collagen cross-linking. GLA-domains are protein regions commonly modified by γ-carboxylation. POSTN harbors 28 vitamin K-dependent GLA-domains in its collagen-binding domain, which is an unusually large number compared to 3 to 5 GLA domains in other matricellular proteins, and the role of GLA-domain γ-carboxylation in this protein is not well understood. POSTN is expressed by myoepithelial cells in normal mammary epithelia. Although myoepithelial cells are lost during aging and breast cancer progression, POSTN is highly expressed by the carcinoma cells and cancer associated fibroblasts (Grignani et al., 1993; Grigoriadis et al., 2006). Preventing POSTN GLA-domain γ-carboxylation and stopping it from exerting its effect as a pro-survival and pro-proliferative protein may constitute a second possible mechanism for warfarin-driven breast cancer prevention.

Additional study of warfarin use in a prevention setting is merited based on the multiple human population and mouse studies showing a putative protective effect. However, contemplating the use of warfarin specifically for cancer prevention raises a number of serious safety challenges, and a better overall understanding of its effects at various doses in epithelial cells and tissue is still needed.

## Metformin for Prevention

Metformin (1,1-dimethylbiguanide hydrochloride) belongs to the biguanide family of oral hypoglycemic agents that are used commonly to treat type II diabetes and insulin resistance. Insulin resistance occurs when peripheral tissues gradually lose their ability to uptake glucose in response to insulin. This provokes the pancreas to produce further insulin and causes elevated serum insulin levels. Metformin lowers serum glucose, increases insulin sensitivity in peripheral tissues and reduces serum insulin levels by a number of mechanisms (Rena et al., 2017). These include reducing hepatic glucose production and inhibiting mitochondrial ATP generation (Owen et al., 2000). Low ATP levels are sensed by AMP-activated protein kinase (AMPK) (Hawley et al., 2010; Rena et al., 2017), which in turn activates signaling pathways to replenish ATP supplies. Simultaneously, AMPK inhibits ATP-consuming synthetic pathways such as gluconeogenesis and lipid synthesis (Carling et al., 2011; Hardie, 2011), thus reducing insulin resistance.

**FIGURE 3:**
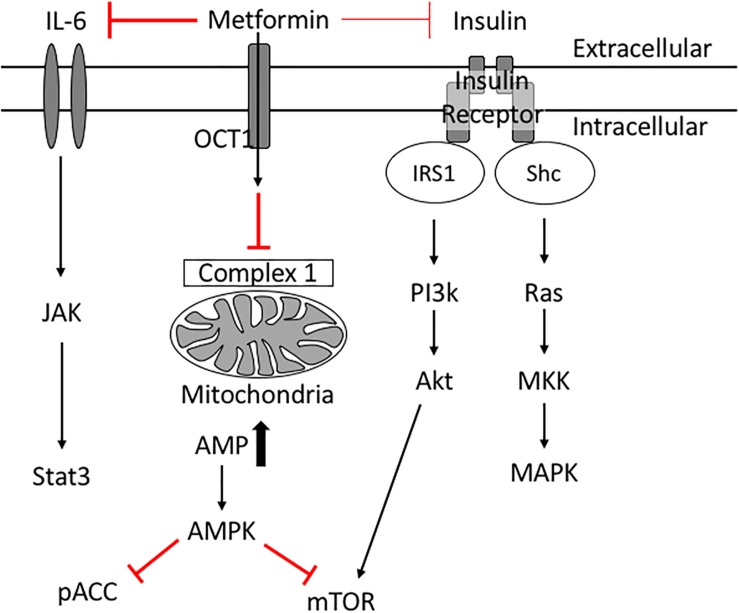
Potential molecular mechanisms of Metformin’s anti-cancer effects. Metformin inhibits complex 1 in the mitochondria thus reducing ATP production. Low levels of ATP activate AMPK which inhibits mTOR. Metformin improves peripheral tissue sensitivity to insulin and reduces insulin levels in the blood. Reduction of downstream signaling through the insulin receptor results in reduction of PI3K/Akt/mTOR signaling as well as RAS/MAPK signaling leading to reduced cellular proliferation. Metformin also induces its cancer preventative effects via inhibiting IL6 mediated activation of JAK/Stat3 signaling involved in tumorigenesis.

Insulin resistance is a key risk factor for age-related breast cancers (Lipscombe et al., 2006; Kabat et al., 2009; Ibarra-Drendall et al., 2011; Gunter et al., 2015; Luque et al., 2017). High insulin levels are positively associated with an increased breast cancer risk in post-menopausal women (Gunter et al., 2015). In addition, women with serum insulin levels in the upper tertile are more than twice as likely to develop breast cancer (Kabat et al., 2009). High indices of insulin resistance are associated also with poor prognosis in women with early and metastatic stages of breast cancer (Gennari et al., 2014; Ferroni et al., 2016). Insulin acts as a breast cancer cell mitogen directly and indirectly via insulin-like growth factors (IGFs) (David and Linda, 2012). When insulin binds its receptor, phosphatidylinositol 3-kinase (PI3K) is activated, which in turn activates Akt/mTOR. Insulin also activates Ras and subsequently mitogen-activated protein kinase (MAPK), inducing cell proliferation and survival (David and Linda, 2012; Figure 3). These studies suggest therapeutics designed to treat insulin resistance may help treat breast cancer in diabetic patients.

Epidemiologic studies revealed that metformin use is associated with decreased cancer and cancer-associated mortality in diabetic patients (Bowker et al., 2006; Jiralerspong et al., 2009; Bodmer et al., 2010; Kim et al., 2018). Diabetic patients on long-term metformin were 56% less likely to develop breast cancer compared with control patients (Bodmer et al., 2010), and had reduced cancer-related mortality (Bowker et al., 2006). At a cellular level, metformin inhibits the growth of breast cancer cells *in vivo* (Zakikhani et al., 2006). Metformin is thought to be anti-neoplastic because it inhibits signaling pathways that fuel breast cancer cell proliferation and protein synthesis. For example, metformin activates AMPK (Hawley et al., 2010; Howell et al., 2017; Rena et al., 2017); activated AMPK inhibits mTOR (Howell et al., 2017) and phospho-Acetyl-CoA carboxylase (pACC) thus leading to suppression of normal and tumor cell growth (Ibarra-Drendall et al., 2011). Metformin’s reduction of insulin levels reduces downstream signaling through the insulin receptor (PI3K/AKT/mTOR) (Zi et al., 2018), and simultaneously reduces signaling to the Ras/MAPK pathway (Ibarra-Drendall et al., 2011; David and Linda, 2012) collectively resulting in reduced cancer cell proliferation and survival. Through these mechanisms metformin has potential beneficial effects in diabetic breast cancer patients.

It is reasonable to speculate that metformin may help non-diabetic breast cancer patients as well by targeting different mechanisms. Indeed, metformin was shown to prevent some aging phenotypes *in vivo* and *in vitro* (Kiho et al., 2005; Diamanti-Kandarakis et al., 2007; Anisimov, 2010; Barzilai et al., 2016). For example, metformin prevents the formation of advanced glycation end products (AGEs) *in vitro*, which normally accumulate in various tissues as a result of aging and long-term diabetes (Kiho et al., 2005; Luevano-Contreras and Chapman-Novakofski, 2010; Vlassara and Uribarri, 2014). Metformin reduced AGE levels in women with polycystic ovary syndrome (characterized by insulin resistance) after a 6-month-long treatment (Diamanti-Kandarakis et al., 2007). Furthermore, metformin limited age-associated senescence in mouse myoblasts (Jadhav et al., 2013) and prevented SASP in human fetal lung fibroblasts (Moiseeva et al., 2013). While it is controversial whether itself affects glucose metabolism and insulin sensitivity (Refaie et al., 2006), especially when accounting for lean body mass, BMI and sex (Chia et al., 2018), there is enough evidence to suggest that hyperinsulinemia levels accelerate aging phenotypes, promote age-related diseases and reduces overall lifespan (Facchini et al., 2000; Johnson and Templeman, 2016). Metformin may slow these processes and improve healthspan by reducing hyperinsulinemia and improving peripheral tissue insulin sensitivity (Martin-Montalvo et al., 2013; Bannister et al., 2014).

Metformin also reduces inflammation associated with insulin resistance, diabetes and aging (Saisho, 2015). Metformin’s anti-inflammatory effects include inhibition of monocyte to macrophage differentiation (Vasamsetti et al., 2015), and inhibition of multiple pro-inflammatory cytokines and related signaling such as IL-6, IL-1β, C-X-C motif ligand 1/2 (CXCL1/2) and NF-κB (Cameron et al., 2016). These effects also were observed in studies of patients with impaired fasting glucose and diabetes (Krysiak and Okopien, 2012, 2013). Reduction in IL-6 levels due to metformin administration was shown to cause a reduction of some cancer stem cells (Iliopoulos et al., 2011). Low doses of metformin selectively killed breast cancer stem cells in four different subtypes of breast cancer (Hirsch et al., 2009).

Thus, current studies suggest a beneficial role for metformin on breast cancer prevention, treatment, and outcome. Indeed, metformin is already being tested in a multicenter clinical trial for its ability to prevent breast cancer in women who exhibit atypical hyperplasia (NCT01905046). Metformin is a relatively inexpensive and safe drug with minimal side effects. The most common side effect is minor gastrointestinal upset, whereas the most serious, yet rare, one is lactic acidosis, especially in patients with renal failure. Collectively, these factors suggest metformin is a worthy drug candidate in the context of breast cancer prevention.

## Patient Advocate Perspectives

### Advocate #1

Notes4Hope.org is a non-profit organization that focuses on healthy lifestyle as a means to prevent breast cancer. There are many chemicals in our terrestrial environment, in our air, in our household and beauty products, and in our foods that have been linked, to one degree or another, to the development of breast cancer. Chemical production in the United States has increased 15-fold since the 1950s, and a number of chemicals that are used in food production and manufacturing exert unintended deleterious biological effects. More research is needed to understand whether there are negative impacts on breast tissue biology of the chemicals used in food production and product manufacturing. Furthermore, education focused on an individual’s incremental and sustainable choices to reduce stress, increase wellness practices, change household and beauty products, and consume more organic and pastured foods can serve as basis for preventing breast cancer. We recognize that diet and lifestyle are intrinsic to culture, and thus conscious changes can be met with significant cultural inertia. Because the panoply of chemicals produced for medical and commercial purposes have as much potential to do harm as they have to heal, the modern pharmacopeia could be used also to augment healthy lifestyle choices. This review considers repurposing medicines like warfarin and metformin, made originally to treat heart disease and diabetes, to prevent breast cancer. While this concept is appealing, as advocates, our excitement should be counterbalanced by the same skepticism with which we view other chemicals used for medicine and manufacturing. Further research should be done to conclude whether or not these medicines do affect breast biology in a positive way, and if they can be used in a manner that does not alter an otherwise healthy aging trajectory.

### Advocate #2

Rethinking the limitations of incremental progress requires new ideas and a collaborative ecosystem across sectors, disciplines, and areas of expertise. Aligning experiential and professionalized expertise and insights, advocates bring unique perspectives to the research table as they lend support, challenge assumptions, inspire change, and assist with responsibly advancing basic science and translational research agendas. Peering into the future of science to improve clinical outcomes, researchers in the LaBarge lab have collaboratively identified innovative cutting-edge scientific ideas on the frontiers of their respective disciplines. Urging cautious optimism within an understanding of cell and tissue biology, they argue that there are some opportunities that we should consider for future prevention targets. Clearly, the public needs awareness regarding emerging new scientific rationales. However, advocates caution that we must not risk fooling ourselves. There does seem to be potential benefits of repurposing anticoagulant drugs such as warfarin or diabetes drugs such as metformin, thus meriting renewed investigation as potential candidates for prevention of breast cancer. Because they act in part by inhibiting tissue-level changes associated with aging, advocates look critically at the value proposition and demand evidence of pill effectiveness and drug safety profiles. If there is insufficient evidence of safety, let us not begin giving the healthy aging population potentially toxic drugs in the name of prevention. As vital catalysts for transdisciplinary innovation, research advocates are thrilled to play a vital role in shaping this effort at study inception. They enthusiastically urge research team members to dive deeper into the scientific as well as the humanistic applications of repurposing drugs as anti-breast cancer agents for the aging population. Moreover, cooperation between researchers and advocates helps encourage team members to speak up about the landscape of uncertainties encountered as they jointly tackle what accounts for the uniqueness of breast cancer prevention in the aging population.

## Discussion

Our intention with this review is to stimulate thinking around how breast cancer prevention might be approached differently by considering the mechanisms driving change in breast tissue that are consequences of aging – the single greatest risk factor for breast cancer. Herein, we examined a continuum from highly theoretical aspects of breast tissue biology that represent potential prevention targets, such as the transition between normal and immortal states, to treatment modalities that are already in some form of clinical deployment. We hypothesized that age-related changes in the tissue may create a susceptible microenvironment for breast cancer progression that can be targeted with drugs for preventing breast cancer. Epidemiological evidence suggests that two existing drugs, warfarin and metformin, typically used for non-cancer diseases, merit renewed investigation as potential candidates for prevention of breast cancer and that they act in part by inhibiting tissue-level changes associated with aging. However, even in our optimism toward the repurposing of these drugs, it must be respected that these drugs (warfarin in particular) can have dangerous side effects. Thus, it will be crucial to understand whether the animal experiments, showing that sub-therapeutic doses of warfarin can exert anti-cancer effects, are safely translatable to humans. If the negative impacts of these decades-old medications cannot be sufficiently mitigated to warrant testing in a normal risk population, then use in high-risk populations could be considered, as is currently the case for metformin. The aging immune system also likely contributes to aging phenotypes that, at the tissue level, contribute to breast cancer and is therefore an important area of research that may provide novel targets for prevention of age-associated breast cancer. The consequences of age-related shifts in the immune system and epithelial-immune cell interactions over a lifetime need to be better understood. Research examining the immortalization barrier to breast cancer progression is in its infancy, but may identify new targets of this rate-limiting step in cancer progression.

## Author Contributions

TF, ML, AZ, SSh, SSa, and SP wrote the different sections of the first draft of the review. TF, ML, AZ, and SSh composed the figures. MS revised the sections of the manuscript and figures. TF and ML composed, revised, and approved the final submission.

## Conflict of Interest Statement

ML and MS have patents that relate to their research in breast cancer prevention. The remaining authors declare that the research was conducted in the absence of any commercial or financial relationships that could be construed as a potential conflict of interest.
